# The Optimal Lowest Instrumented Vertebra to Prevent the Distal Adding-On Phenomenon in Patients Undergoing Selective Thoracic Fusion for Adolescent Idiopathic Scoliosis with Lenke Type 1A and 1B Curves: Comparison of Nine Selection Criteria

**DOI:** 10.3390/jcm13133859

**Published:** 2024-06-30

**Authors:** Se-Jun Park, Jin-Sung Park, Dong-Ho Kang, Chong-Suh Lee

**Affiliations:** 1 Department of Orthopedic Surgery, Spine Center, Samsung Medical Center, Sungkyunkwan University School of Medicine, Seoul 06351, Republic of Korea; sejunos@gmail.com (S.-J.P.); paridot@hanmail.net (J.-S.P.); 2 Department of Orthopedic Surgery, Haeundae Bumin Hospital, Busan 48094, Republic of Korea; csl3503@gmail.com

**Keywords:** adolescent idiopathic scoliosis, distal adding-on, lowest instrumented vertebra, selection of level, substantially touching vertebra

## Abstract

**Background/Objectives**: There is no solid consensus regarding which lowest instrumented vertebra (LIV) selection criterion is best to prevent distal adding-on (DA) after adolescent idiopathic scoliosis (AIS) surgery. This study aims to search out the LIV selection criteria in the literature and to compare the ability of each LIV selection criterion to prevent DA in patients with AIS. **Methods**: Patients who underwent thoracic fusion for AIS of Lenke type 1A or 1B were included in this study. Nine criteria for LIV selection were found in a literature review. For each patient, whether the postoperative actual location of LIV was met with the suggested locations of the LIV was assessed. The preventive ability of nine criteria against DA was evaluated using logistic regression analysis. The patients who met the LIV selection criteria but developed DA were investigated. **Results**: The study cohort consisted of 145 consecutive patients with a mean age of 14.8 years. The criteria of Suk (OR = 0.267), Parisini (OR = 0.230), Wang (OR = 0.289), and Qin (OR = 0.210) showed a significantly decreased risk of DA if the LIV selection criterion was chosen at each suggested landmark. As the additional levels were fused, there was no statistically significant benefit in further reducing the risk of DA. Among the patients who met each criterion, the incidence of DA was lower in criteria by Takahashi (5.9%), Qin (7.1%), and King (7.4%) than the others. **Conclusions**: Qin’s criterion, using the substantially touching vertebra concept, has the highest preventive ability against DA development. Extending the instrumentation further distal to this suggested LIV criterion did not add further benefit.

## 1. Introduction

Thoracic fusion is a common strategy used for surgical treatment for adolescent idiopathic scoliosis (AIS) with Lekne type 1A and 1B curves, with the goals of achieving surgical correction of thoracic curves while preserving as many lumbar motion segments as possible [1,2,3,4,5,6]. In thoracic fusion for these curves, the distal adding-on (DA) phenomenon, which is characterized as a progressive correction loss due to an increase in vertebral deviation or disc angulation below the lowest instrumented vertebra (LIV), has been a concern for a long time. Postoperative DA can negatively affect the clinical outcomes and sometimes necessitates revision surgery [7]. DA sometimes leads to increased coronal decompensation, which could lead to a bad appearance and degenerative changes later in life [5]. Numerous studies have demonstrated that improper location of the LIV is strongly associated with DA development; therefore, proper selection of the LIV level is warranted to prevent DA [7,8,9,10,11,12,13,14,15,16,17]. 

With regard to LIV determination, several authors have proposed the optimal LIV location on the basis of diverse radiographic parameters such as the location within the stable zone, vertebral rotation, adjacent disc angulation, and curve flexibility [10,11,12,13,18,19]. Since King et al. first suggested that the optimal UIV level should be stable vertebra (SV), nine LIV selection criteria in Lenke type 1 curves have been proposed in the literature so far [7,10,11,12,13,14,15,16,18]. Although each criterion demonstrated its preventive ability against DA development in their original articles, they still require the external validation with regard to DA prevention. In addition, because each criterion was suggested by different radiographic findings, each criterion may provide different optimal LIV levels even for one patient. Therefore, we thought it necessary to comprehensively evaluate the preventive ability against DA development including all the current criteria for Lenke 1A and 1B curves. 

Thus, this study primarily aims to compare the ability of each LIV selection criterion to prevent DA in patients with AIS of Lenke type 1A and 1B curves. We also investigated how the optimal LIV levels suggested by the nine criteria are agreeable with each other.

## 2. Materials and Methods

### 2.1. Study Cohort

This study was approved by the institutional review board at our institution (SMC 2022-08-128), and the need for informed consent was waived because of the retrospective nature of this study. The consecutive patients who underwent posterior thoracic fusion for AIS with Lenke type 1A or 1B curves between 2012 and 2020 were included. The minimum follow-up duration was two years. Exclusion criteria are as follows: patients with non-idiopathic scoliosis, patients undergoing anterior thoracic surgery, and patients with a follow-up loss within two years.

### 2.2. Surgical Protocol

Surgeries were performed by one of the two senior surgeons using conventional pedicle screw-based posterior instrumentation and fusion. The surgical techniques used to correct deformity were the same for all patients. After inserting pedicle screws, the rod was inserted on the concave side. The scoliosis was corrected, and thoracic kyphosis was created by rotating the rod counterclockwise. Direct vertebral rotation was performed at the 3–4 vertebrae of the apex, and then the screw caps were locked. A convex rod was inserted with underbending to press the thoracic hump, and segmental compression and distraction were performed around the UIV and LIV areas to correct the remnant vertebral tilt. The LIV was selected on a standing whole-spine radiograph considering various radiographic parameters such as SV, neutral vertebra (NV), end vertebra (EV), and their relationship to the center sacral vertical line (CSVL). 

### 2.3. Searching for the LIV Selection Criteria

Published clinical studies and review articles dealing with the selection of LIV in AIS for surgical treatment were included for this study. PubMed, Scopus, Web of Science, and Google Scholar were used to search relevant studies. The following key words were searched in the databases: “adolescent idiopathic scoliosis”, “fusion level”, “lowest instrumented vertebra”, “adding-on”, “Lenke type 1”, “Lenke type 1A or 1B”, and various combinations of these terms. A total of 57 studies were identified. Finally, nine articles suggested LIV selection criteria for Lenke type 1 curves [7,10,11,12,13,14,15,16,18]. The nine criteria of LIV selection are summarized in Table 1.

### 2.4. Radiographic Measurements

**Figure 1:**
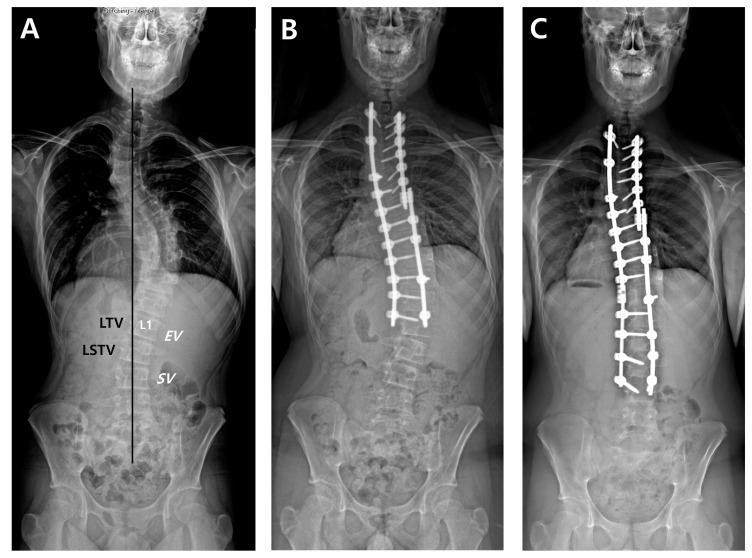
A thirteen-year-old male underwent posterior fusion from T1 to L1 for AIS of Lenke type 2A curves (**A**,**B**). Revision surgery with fusion extension to L3 was carried out due to the development and progression of DA (**C**). According to the nine LIV selection criteria, the postoperative actual LIV (L1) met the criteria by Wang, Sarlak, Matsumoto, and Fischer. The location of the postoperative LIV was proximal to the suggested LIVs by King (L3), Suk (L3), Parisini (L2), Takahashi (L4), and Qin (L2).

For all patients, whole-spine standing posteroanterior, lateral, and fulcrum bending radiographs were taken preoperatively. After surgery, standing posteroanterior and lateral whole-spine radiographs were performed at six weeks, three months, six months, and then annually as part of a routine evaluation of all scoliosis cases. First, we designated radiographic parameters such as EV, NV, and SV on a preoperative whole-spine AP radiograph (Figure 1). The last touching vertebra (LTV) and last substantially touching vertebra (LSTV) were measured using CSVL. On the same radiograph, the nine suggested LIVs according to the selection criteria were separately recorded. Then, we compared the location of suggested LIVs and the actual LIV, and evaluated whether the actual LIV was the same, proximal, or distal relative to the suggested LIV. DA was defined as a progressive increase in the number of vertebrae included within the distal curve, with either an increase of more than 5 mm in the deviation of the first vertebra below the instrumentation from the CSVL or an increase of more than 5° in the angulation of the first disc below the instrumentation [7]. Finally, the preoperative Cobb’s angle (CA) of the main thoracic curve (MTC), flexibility of the MTC, postoperative CA of the MTC, and correction rate of the MTC were measured. 

### 2.5. Outcomes Measures

We calculated the mean level and distribution of the suggested LIVs according to the nine criteria. For describing LIV location, vertebral bodies were numbered as follows: 9 as T9, 10 as T10, 11 as T11, 12 as T12, 13 as L1, 14 as L2, 15 as L3, and 16 as L4. Then, we evaluated the preventive capability against DA separately when the actual LIV was proximal to the suggested LIV location and when the actual LIV was distal to the suggested LIV location. We also analyzed the degree of agreement among the nine criteria of LIV selection in pairs. Finally, we investigated the patients who met the LIV selection criteria but developed DA in each criterion.

### 2.6. Statistical Analysis

Data were presented as frequencies with percentages for categorical variables and means with standard deviations for continuous variables. The Chi-square test or Fisher’s exact test were performed to compare the categorical variables and analysis of variance (ANOVA) for continuous variables among groups. Logistic regression analysis was performed to determine the preventive capability of DA. The results were expressed as the odds ratio (OR) with a 95% confidence interval (CI). Agreement among the nine criteria was evaluated using the intra-class correlation (ICC) test. ICC values are interpreted as follows: >0.8 denotes very good; 0.6~0.8, good; 0.4~0.6, moderate; 0.2~0.4, fair; and <0.2, poor. Statistical analyses were performed by professional statisticians using SPSS (version 27.0.0; IBM Corp., Armonk, NY, USA). A *p* value of < 0.05 was considered statistically significant.

## 3. Results

### 3.1. Baseline Data

The study cohort consisted of 145 consecutive AIS patients (26 males and 119 females). The mean age at the time of surgery was 14.8 ± 2.6 years. There were 117 patients with Lenke type 1A and 28 with type 2A. Preoperative CA of the MTC was 55.1 ± 10.3°, and flexibility of the MTC was 52.7 ± 17.2%. Postoperative CA of the MTC improved to 52.7 ± 17.2° with a correction rate of 65.1 ± 13.3%. There were 54 patients who met the criteria by King, 109 by Suk, 118 by Parisini, 117 by Wang, 136 by Sarlack, 17 by Takahashi, 109 by Matsumoto, 84 by Qin, and 112 by Fischer (Table 2). There were no significant differences with regard to female sex, age, Lenke type, preoperative CA of the MTC, flexibility of the MTC, postoperative CA of the MTC, and correction rate among the patients who met the nine criteria (Table 2).

### 3.2. Suggested LIV Location According to the Criteria

**Figure 2:**
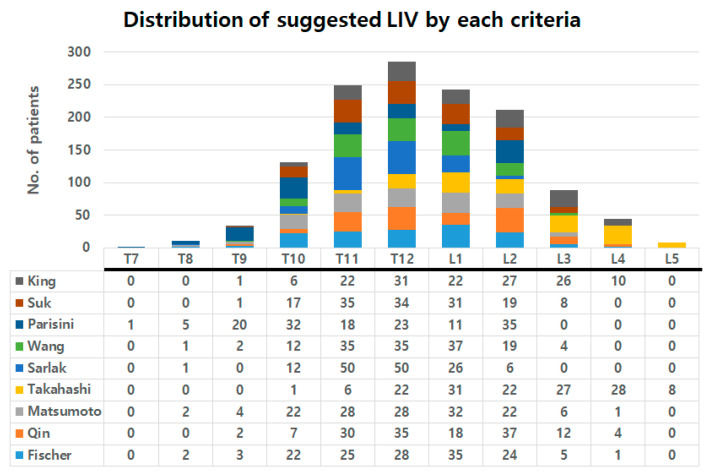
Graph showing distribution of suggested LIV by each criterion.

The suggested location of LIV was the most proximal in Parisini’s criteria (mean level of 11.4), while the LIV location suggested by Takashahi was the most distal (mean level of 14.1) (Table 3). The LIVs suggested by the criteria were most frequent in T12, followed by T11 and L1. The detailed distributions of LIV suggested by each criterion are also presented in Figure 2. The actual location of LIV was the mean level of 12.3 ± 1.1; the mean level of EV was 11.7 ± 1.0, NV was 12.3 ± 1.6, and SV was 13.1 ± 1.7 (Table 3). The mean actual LIV level of the current study population was approximately similar to the NV level and was located between the EV and SV levels.

### 3.3. Comparison of Preventive Ability against DA by Odds Ratio

During a mean follow-up duration of 82.4 ± 58.6 months, a total 24 of 145 patients (16.6%) developed DA. The rates of DA did not differ according to the LIV (*p* = 0.241). In logistic regression analysis, when the actual LIV location is same as the LIV suggested by the criteria of Suk, Parisini, Wang, and Qin, the risk of DA significantly decreased compared to cases with stopping at a proximal level to the suggested LIVs (OR = 0.267, *p* = 0.010 for Suk; OR = 0.230, *p* = 0.045 for Parisini; OR = 0.289, *p* = 0.020 for Wang; and OR = 0.210, *p* = 0.004 for Qin) (Table 4). The data also showed that even if levels were fused for longer than the suggested LIV location, there was no statistically significant benefit in further reducing the risk of DA except for Fischer’s criteria. In the case of Fischer’s criteria, the risk of DA was reduced when selecting a level distal to rather than at the suggested LIV level (OR = 0.221, *p* = 0.025).

### 3.4. Agreement of LIV among the Nine LIV Selection Criteria

All criteria of LIV selection showed various degrees of correlation in pairs with different agreement powers (Table 5). King and Takahashi (ICC = 0.850), King and Qin (ICC = 0.916), Parisini and Matsumoto (ICC = 0.827), Wang and Matsumoto (ICC = 0.872), Wang and Qin (ICC = 0.833), Wang and Fischer (ICC = 0.851), Matsumoto and Qin (ICC = 0.885), Matsumoto and Fischer (ICC = 0.960), and Qin and Fischer (ICC = 0.871) showed very good agreement.

### 3.5. Development of DA despite Suggested LIV Criteria Being Met

**Figure 3:**
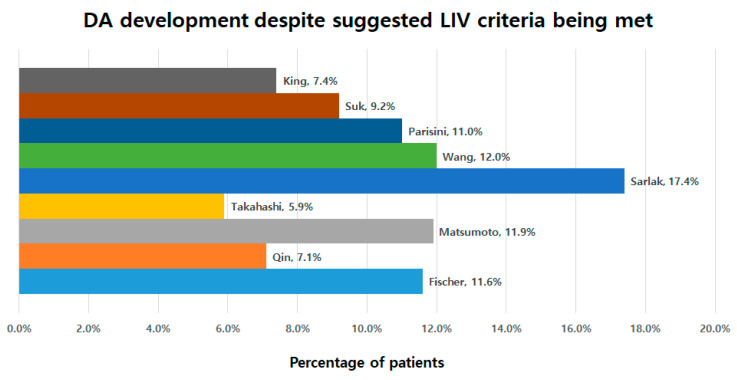
The incidence of DA despite the suggested LIV criteria being met.

The number of patients who developed DA despite the suggested LIV criteria being met were evaluated. There were 7.4% of such patients (4/54) for King, 9.2% (10/109) for Suk, 11.0% (13/118) for Parisini, 12.0% (14/117) for Wang, 14.7% (20/136) for Sarlak, 5.9% (1/17) for Takahashi, 11.9% (13/109) for Matsumoto, 7.1% (6/84) for Qin, and 11.6% (13/112) for Fischer (Figure 3).

## 4. Discussion

The ultimate goal for AIS correction is to achieve a globally balanced spine in both the coronal and sagittal planes while maintaining as many motion segments as possible. Despite promising results after fusion surgery for AIS, there is always a trade-off between curve correction and loss of mobility. Therefore, we believe that it is necessary to address mobility loss. Helenius et al. [20]. studied the spinal mobility and functional outcomes in 78 patients who underwent fusion surgery using Harrington instrumentation. With use of a goniometer, they found decreased lumbar flexion in 18 patients (23%), decreased lumbar extension in 22 patients (28%), and decreased trunk side-bending in 46 patients (59%). However, they concluded that patients could perform, on average, as well as the reference population in nondynamometric trunk strength tests, including sit-ups, arch-ups, and squatting. Sanchez et al. [21]. performed a similar study on trunk flexibility using a dual digital inclinometer. They divided patients into three groups based on the LIV level: group 1 (T12, L1, or L2), group 2 (L3), and group 3 (L4, L5, or S1). They observed that group 3 showed a significantly decreased trunk flexibility compared with the other groups. Despite a few reports comparing the quality of life according to fusion levels, these two studies indirectly support the plausible advantages of short fusion on a better quality of life.

Although deformity correction seems to be easy in Lenke type 1A or 2A curves, there has been a major concern of DA. It is well known that determination of the spinal fusion level plays an important role in preventing DA. Despite the importance of LIV selection, there is no universally accepted method for determining the LIV for AIS correction. In the present study, we found nine criteria for the determination of LIV to prevent the DA phenomenon (Table 1). 

In this study, we compared the relations of LIV location between the nine criteria and the current study population (Table 3). The LIVs suggested by the criteria were most frequent in T12, followed by T11 and L1. This seems to be mainly where thoracic fusion was conducted for the cases of Lenke 1A or 2A curves. In our study, the mean LIV was 12.3 ± 1.1, which was approximately similar to the NV level, and between the EV and SV levels. The suggested LIVs somewhat differ according to the nine criteria. The LIVs suggested by King and Takahashi et al. were more distal than the others. On the other hand, Parisini and Sarlak’s criteria suggested a more proximal level than the others. That may be because King and Takahashi’s criteria are based on the SV and Parisini and Sarlak’s criteria are based on the EV [11,12,13,18].

Among the nine criteria, we found four criteria (by Suk, Parisini, Wang, and Qin) that showed a statistically significant preventive ability when compared to the case of short fusion relative to the suggested LIVs (Table 4). Initially, in 2003, Suk et al. suggested that the curve should be fused down to the NV when the preoperative NV and EN showed no more than two level gap differences. When the gap was more than two levels, fusion down to NV-1 was recommended [10]. In 2009, Parisini et al. recommended that if the rotation of the first vertebra just below the EV is in the same direction as the thoracic curve, and if the SV and EV have a difference of >2 levels, fusion should extended to L2 or L3. Otherwise, SV-2 or SV-3 can be selected as the LIV. These two criteria were significantly preventive against DA development, with an OR of 0.267 for Suk’s criteria and 0.230 for Parisini’s criteria. Although the EV, NV, and SV have been the traditional guide for LIV selection, the recent literatures have pointed out the low measurement reliability for locating these levels, which limits the application of these parameters chosen to be the LIV [7,14,16,22]. To overcome the inconsistencies of using EV, NV, and SV as methods of LIV selection, several authors have demonstrated that using the last touching vertebra (LTV) and substantially touched vertebrae (STV) are more reliable methods of LIV selection for Lenke 1 and 2 curves [7,14,15,16]. Therefore, Wang et al. recommended choosing the first vertebra cranially that deviates from the CSVL >10 mm as the LIV [7]. In the present study, Wang’s criteria significantly prevented against DA, with an OR of 0.289. In 2013, the concept of LTV was first introduced by Matsumoto et al., which is defined as the last cephalad vertebra touched by the CSVL. They suggested choosing the LIV as at least level to the LTV to avoid postoperative DA [14]. However, in our study results we found that using the LTV as the LIV, which is one of the commonly used landmarks for AIS correction, did not significantly prevent DA. In 2016, Qin et al. developed and subdivided the LTV concept because they realized that the original LIV concept was insufficient to predict the DA phenomenon. They defined the STV as the vertebra where the CSVL was between the pedicles or touching the pedicle and the non-substantially touched vertebrae (nSTV) as the vertebra where the CSVL was only touching the corner of the vertebra lateral to the pedicle. The authors recommended choosing the STV or nSTV+1 as the LIV for patients with Lenke type 1A curves [16]. Our data showed that Qin’s criteria had the ability to prevent the occurrence of DA, with an OR of 0.210, which was the lowest OR among the four statistically significant criteria. On the other hand, it is interesting that fusion beyond the suggested LIV did not provide a further statistically significant risk reduction in DA in all criteria except for Fischer’s criterion. In the case of Fischer’s criterion, a more distal fusion than suggested was statistically significant in preventing DA (OR = 0.221). In other words, it seemed that Fischer’s criteria suggesting LTV within NV-2 is too short to prevent DA. 

Because all criteria have been introduced using different landmarks for the one goal of preventing DA, it is necessary to assess how each LIV suggested by the nine criteria matches each other. We found that all criteria had a various degree of agreement in pairs (Table 5). King and Takahashi (ICC = 0.850) and King and Qin (ICC = 0.916), based on a similar concept of SV or bisecting vertebra bodies, had very good agreement. Also, Wang, Matsumoto, Qin, and Fischer all had very good agreement with each other (ICC > 0.8). This is because these four criteria are based on a concept similar to LTV.

Finally, it is necessary to determine which criterion is best in LIV selection against the development of DA. For being the ideal criteria, the following conditions would be met: (1) a statistically proven ability to prevent DA, (2) a not-too-distal location of suggested LIV, and (3) less patients who developed DA when the suggested LIV criteria are met. In a logistic regression analysis, Qin’s criteria (OR = 0.210) had the highest prevention rate in reducing the DA among the four significant criteria (Table 4). The suggested location of LIV in most criteria was similar around T12, except for King and Takahashi’s criteria, which suggest a more distal location of LIV (Table 3). In terms of DA development despite the suggested LIV criteria being met, the incidence rate of those patients was lower for the criteria by Takahashi (5.9%), Qin (7.1%), and King (7.4%) compared to other criteria (Figure 3). When these results are taken into account, we can suggest that Qin’s criterion would be the best one because it showed the lowest prevention rate, with a not-too-distal location and a low incidence rate of DA despite the suggested LIV criteria being met.

We have to acknowledge a few limitations. First, a small sample size in a single institution and the retrospective nature of this study are inherent limitations. Second, not all criteria may be applied to every case, likely rendering comparison difficult. However, that point, in turn, means that different criteria can be applied even for a single patient. In addition, there might be confounding factors between groups with different LIV selection criteria, such as different Cobb’s angles, potentially leading to bias in the current findings. Third, we did not consider the sagittal plane in radiographs, although the importance of the sagittal plane has been reported in AIS patients recently [23,24,25,26,27,28,29,30]. Therefore, the relation between the development of DA and the sagittal plane needs further research. Finally, our findings could be biased by the relatively small number of patients with DA. Multicenter study is warranted for a sound conclusion.

## 5. Conclusions

Nine LIV selection criteria were compared in terms of their preventive ability against DA. Qin’s criterion using the STV or nSTV+1 has the highest preventive ability against DA development. Extending the instrumentation further distal to this landmark may not be necessary.

## Figures and Tables

**Table 1:** Summary of the nine LIV selection criteria.

Study	Curve Type	No. of Patients	Mean Age (Years)	Mean FU (Years)	Suggestion
King et al. (1983) [18]	King 3, 4, 5	405	14.8	4.0	SV
Suk et al. (2003) [10]	King 3, 4	42	15.5	4.2	Diff. between NV and EV: ≤1 levels → NV ≥2 level → NV-1
Parisini et al. (2009) [11]	Lenke 1A	31	16.3	Min. 2	Direction of rotation at LEV+1 is equal to that of thoracic curve and diff. between SV and EV is ≥3 level → L2 or L3. Otherwise → SV-2 or SV-3
Wang et al. (2011) [7]	Lenke 1A	45	-	3.6	1st vertebrae >10 mm from CSVL
Sarlak et al. (2011) [12]	Lenke 1A	36	15.8	4.3	L3 tilt (−) → LEV-1 L3 tilt (+) → LEV
Takahashi et al. (2011) [13]	Lenke 1B, 1C, 3C	172	14	2	SV+1
Matsumoto et al. (2013) [14]	Lenke 1A	112	16.1	3.6	LTV
Qin et al. (2016) [16]	Lenke 1A	104	14.5	Min. 2	LSTV or nSTV+1
Fischer et al. (2018) [15]	Lenke 1, 2	544	14.7	4.1	LTV within NV-2

SV indicates stable vertebra; NV, neutral vertebra; EV, end vertebra; Diff., difference; Min., minimum; LEV, lower end vertebra; CSVL, center sacral vertical line; LIV, lower instrumented vertebra; LTV, last touched vertebra; LSTV, last substantially touched vertebra; nSTV, non-substantially touched vertebra.

**Table 2:** Comparison of characteristics in patients who met each LIV criterion.

	King	Suk	Parisini	Wang	Sarlak	Takahashi	Matsumoto	Qin	Fischer	*p*
No. of patients	54	109	118	117	136	17	109	84	112	-
Female (%)	83.3%	80.7%	82.2%	82.9%	82.4%	88.2%	81.7%	82.1%	83.9%	0.999
Age (years)	14.5	14.9	14.7	14.7	14.9	13.2	14.7	14.6	14.6	0.536
Lenke 1A (%)	81.5%	79.8%	79.7%	79.5%	80.1%	85.1%	78.9%	77.4%	78.6%	0.770
Pre CA (°)	54.8°	56.1°	56.4°	55.5°	56.0°	52.9°	55.3°	54.9°	55.1°	0.913
Flexibility (%)	53.0%	51.6%	50.7%	51.9%	51.8%	53.9%	52.4%	52.3%	52.7%	0.993
Post CA (°)	19.2°	20.2°	20.6°	19.9°	19.8°	15.1°	19.8°	20.0°	19.5°	0.653
Correction rate (%)	65.3%	64.5%	63.9%	64.7%	65.2%	71.1%	64.7%	64.0%	65.1%	0.775

Numbers are presented as the mean value. LIV indicates lowest instrumented vertebra; pre, preoperative; CA, Cobb’s angle; post, postoperative.

**Table 3:** Summary of suggested LIV location according to the nine criteria.

	Mean ± SD	Minimum	Maximum
*Nine criteria*			
King	13.1 ± 1.7	9.0	16.0
Suk	12.1 ± 1.4	9.0	15.0
Parisini	11.4 ± 1.9	7.0	14.0
Wang	12.1 ± 1.4	8.0	15.0
Sarlak	11.7 ± 1.0	8.0	14.0
Takahashi	14.1 ± 1.7	10.0	17.0
Matsumoto	12.0 ± 1.6	8.0	16.0
Qin	12.6 ± 1.6	9.0	16.0
Fischer	12.1 ± 1.6	8.0	16.0
*Current Study population*			
EV	11.7 ± 1.0	8.0	14.0
NV	12.3 ± 1.6	9.0	16.0
SV	13.1 ± 1.7	9.0	16.0
Postoperative actual LIV	12.3 ± 1.1	9.0	14.0

7 indicates T7; 8, T8; 9, T9; 10, T10; 11, T11; 12, T12; 13, L1; 14, L2; 15, L3; 16, L4; 17, L5; SD, standard deviation; LIV, lowest instrumented vertebra; EV, end vertebra; NV, neutral vertebra; SV, stable vertebra.

**Table 4:** Prevention of distal adding-on according to the nine LIV selection criteria.

	B	S.E	Wald	*p*	Odds Ratio	95% CI
King						
Proximal to LIV vs. at LIV	−1.190	0.653	3.323	0.068	0.304	0.085–1.094
At LIV vs. distal to LIV	−0.251	1.195	0.044	0.833	0.778	0.075–8.095
Suk						
Proximal to LIV vs. at LIV	−1.319	0.513	6.608	**0.010**	0.267	0.098–0.731
At LIV vs. distal to LIV	−1.487	0.816	3.324	0.068	0.226	0.046–1.118
Parisini						
Proximal to LIV vs. at LIV	−1.471	0.734	4.012	**0.045**	0.230	0.054–0.969
At LIV vs. distal to LIV	−0.306	0.705	0.188	0.665	0.736	0.185–2.935
Wang						
Proximal to LIV vs. at LIV	−1.240	0.533	5.407	**0.020**	0.289	0.102–0.823
At LIV vs. distal to LIV	−0.413	0.592	0.486	0.486	0.662	0.208–2.111
Sarlak						
Proximal to LIV vs. at LIV	−1.463	0.754	3.766	0.052	0.231	0.053–1.015
At LIV vs. distal to LIV	−0.138	0.484	0.081	0.775	0.871	0.337–2.251
Takahashi						
Proximal to LIV vs. at LIV	−0.784	1.704	0.533	0.465	0.457	0.056–3.745
At LIV vs. distal to LIV	−1.131	0.229	1.487	0.222	−0.323	0.052–1.985
Matsumoto						
Proximal to LIV vs. at LIV	−0.768	0.501	2.348	0.125	0.464	0.174–1.239
At LIV vs. distal to LIV	−1.162	0.689	2.844	0.092	0.313	0.081–1.207
Qin						
Proximal to LIV vs. at LIV	−1.563	0.544	8.241	**0.004**	0.210	0.072–0.609
At LIV vs. distal to LIV	−0.611	1.125	0.295	0.587	0.543	0.060–4.922
Fischer						
Proximal to LIV vs. at LIV	−0.135	0.507	0.071	0.790	1.145	0.424–3.092
At LIV vs. distal to LIV	−1.511	0.674	5.020	**0.025**	0.221	0.059–0.828

Bold *p* values mean statistical significance. LIV indicates lowest instrumented vertebra; CI indicates confidential interval.

**Table 5:** Agreement of LIV among the nine LIV selection criteria.

	King	Suk	Parisini	Wang	Sarlak	Takahashi	Matsumoto	Qin	Fischer
King	1	0.614	0.621	0.727	0.487	**0.850**	0.789	**0.916**	0.776
Suk		1	0.604	0.710	0.678	0.406	0.710	0.690	0.778
Parisini			1	0.698	0.561	0.422	**0.827**	0.673	0.786
Wang				1	0.720	0.477	**0.872**	**0.833**	**0.851**
Sarlak					1	0.295	0.706	0.611	0.681
Takahashi						1	0.534	0.682	0.527
Matsumoto							1	**0.885**	**0.960**
Qin								1	**0.871**
Fischer									1

Values of mean ICC (intra-class correlation coefficient). Bold *p* values indicate very good agreement among two criteria. Interpretation of ICC value: >0.8 (very good), 0.6~0.8 (good), 0.4~0.6 (moderate), 0.2~0.4 (fair), and <0.2 (poor).

## Data Availability

Data used in this study can be shared upon the reasonable request from the journal. However, it can be limited due to patient privacy and ethical restriction.
